# Supplementary material for: Prevalence and risk factors for feather-damaging behavior in psittacine birds: Analysis of a Japanese nationwide survey
Source: PLoS One. 2021 Jul 14;16(7):e0254610. doi: 10.1371/journal.pone.0254610 (PMC8279392; doi:10.1371/journal.pone.0254610)
Supplement: S1 File — (PDF) [file pone.0254610.s001.pdf]

Informed consent

The purpose of this study is to investigate the relationship between the environment or husbandry and problem behavior of birds.

This study is being conducted by Kazumasa Ebisawa [1. College of Bioresource Sciences, Nihon University, 2. Yokohama bird clinic].

Participation in this study is completely voluntary. If you decide not to participate there will not be any negative consequences. Please be aware that if you decide to participate, you may stop participating at any time and you may decide not to answer any specific question.

Participating in this study may not benefit you directly, but it will help us learn about problem behaviors.

The information you will share with us if you participate in this study will be kept completely confidential to the full extent of the law.

When the study is completed and the data have been analyzed, the list linking participant's name and e-mail address to study numbers will be destroyed.

By submitting this form you are indicating that you have read the description of the study, are over the age of 18, and that you agree to the terms as described.

If you have any questions about this study, or would like a copy of this consent letter please contact [Kazumasa Ebisawa, phone: 045-453-3010, e-mail: yokohamabirdclinic1997@gmail.com].

* 1. I agree to participate in the research study. I understand the purpose and nature of this study and I am participating voluntarily. I understand that I can withdraw from the study at any time, without any penalty or consequences.
○Yes
○No

* 2. I grant permission for the data generated from this questionnaire to be used in the researcher's publications on this topic.
○Yes
○No

* 3. Please type your name in the box below to indicate agreement to participate in this study.
