# Supplementary material for: Prevalence and risk factors for feather-damaging behavior in psittacine birds: Analysis of a Japanese nationwide survey
Source: PLoS One. 2021 Jul 14;16(7):e0254610. doi: 10.1371/journal.pone.0254610 (PMC8279392; doi:10.1371/journal.pone.0254610)
Supplement: S2 File — (PDF) [file pone.0254610.s002.pdf]

Descriptions of the terms used for each behavior

Table A. Descriptions of feather-damaging behavior

Behavior	Description
Feather picking<br>Feather plucking	The bird plucks its feathers with its beak, resulting in loss of the feathers and occasionally injuries to the skin.
Feather chewing	The bird chews its feathers with its beak, resulting in damage to the feathers.
Feather biting	The bird bites its rachis and barb with its beak, resulting in break to the feathers.

Table B. Descriptions of stereotyped behavior

Behavior	Description
Excessive self-grooming	The bird grooms itself frequently and continuously.
Incessant screaming	The bird makes a sharp and continuous vocalization.
Wire chewing	The bird gnaws repeatedly on the wire bars of the cage. While gnawing, individual parrots may pull violently on the wire, making a snapping sound. These movements involve identical body postures or identical locations within the cage.
Sham chewing	The bird makes chewing movements with nothing in its mouth.
Beak rubbing	The bird frequently rubs their beaks on the perch or the wire.
Food manipulation	The bird picks up a food item in the mouth. The food item is not chewed but is instead turned around in the mouth repetitively.
Wing flapping	The bird flaps their wings many times on the perch and the sidewall of the cage.
Pacing	The bird walks back and forth across the perch, turning around upon reaching either end of the perch. Alternatively, the parrot faces the front of the cage and side stepped from one end of the perch to the other. Pacing can be performed along the entire length of the perch or just for a few steps.
Perch circles	The bird walks the length of the perch, climbs up the sidewall of the cage, climbs across the top of the cage, down the opposite sidewall to the perch, completing a vertical circle across the top of the cage and down the sidewall.
Corner flips	The bird turns in small circles in the top corner of the cage.
Route tracing	The bird walks and/or climbs a repeated identical route around the cage.

Table C. Descriptions of reproductive behavior

Behavior	Description
Courtship behavior	Courtship includes wing flapping, head nodding, regurgitation, singing and intricate stepping. The male dances for other birds (generally female) or humans.
Copulation behavior	The male tries to mate with other birds (generally female), or human hands, feet and other parts. The female crouches into a receptive posture in response to stimuli from the male or human hands.
Nesting	The bird looks for and occupies places where it can build the nest, such as the corner of the cage, the bookshelf, the closet and trash cans. The bird sometimes builds the nest using paper, other materials, or its feathers.

Table D. Descriptions of reproductive behavior

Behavior	Description
Vocalization	The bird vocalizes excessively when the owner leaves home.
Locomotor activity	The bird starts getting fidgety and pacing or wing flapping when the owner leaves home.
Decreased appetite	The bird eats little or no food during the absence of its owner.
Destructiveness	The bird bites or destroys perches or toys excessively during the absence of its owner.
