# Supplementary material for: Prevalence and risk factors for feather-damaging behavior in psittacine birds: Analysis of a Japanese nationwide survey
Source: PLoS One. 2021 Jul 14;16(7):e0254610. doi: 10.1371/journal.pone.0254610 (PMC8279392; doi:10.1371/journal.pone.0254610)
Supplement: Attachment — Submitted filename: Ref report for Japanese FDB paper.docx [file pone.0254610.s005.docx]

This paper is well-written, and based on a very nice dataset that is also well-analysed.

**METHODS**

Did you need human ethics approval, for surveying owners? (In my country one would need that)

Re identifying species, any idea how accurate owners were? Was doubt here a reason why “Some of the congeners or closely

related species were grouped together because of their similar prevalence of FDB”.? (Lines 121 – 123).

Also then in Tables 4 - 6, the term “species” does not really mean “species” then? If not it should be changed to some else (e.g. “Species/genus”??)

Lines 122- 124: “Species with a proportion of less than 2% were excluded to clarify the trends in FDB according to species or groups”. I understand this is because you want to ID the risk factors for FDB, but can you clarify whether your overall prevalence value of 11.7% was calculated before or after this exclusion?

Lines 146-147: “The forward selection method based on the likelihood ratio was used to select the variables”.: can you give more details so that someone else could replicate what you did?

**RESULTS**

Table 5: The interactions need explaining: it’s great that you ran them, but it's not enough to just present the P values – please let the reader know what the pattern was that lead to each significant interaction. Also is one of the two “bird sex” column really “bird age”?

Furthermore, where an interaction between terms A and B is significant, that means it’s not OK to talk about any significant main effects of terms A or B, because they probably are just driven by the interaction. This has considerable interactions for the Discussion.

**DISCUSSION**

This is interesting and well written, but needs work because it presents effects of “species” (really “species and genus”), “age” and other effects are presented as though they are simple main effects, when really they have interactive effects so such an interpretation is not valid. For example, species interacts with 8 other terms (see Table 5), which means that effect of species varies in magnitude with these 8 factors (it may hold for some levels of these but not for other). Likwise bird age interacts with three or more other factors (hard to tell because of a possible typo in the columns names of Table 5), such that there is no general pattern of bird age – it varies according to all these other factors.

Line 217 – “for companionship as a substitute for children": isn’t this rather sexist? Maybe older women are just very skilled at keeping animals an enjoy it?

Line 294 “ acquisition source showed only a weak interaction with separation anxiety (Cramer’s V = 0.097, P < 0.001).” Isn’t a P that small indicative of a rather strong interaction? Also what does this interaction mean i.e. what causes it? (Again more information needs to be provided in the Results).
