# Supplementary material for: Prevalence and risk factors for feather-damaging behavior in psittacine birds: Analysis of a Japanese nationwide survey
Source: PLoS One. 2021 Jul 14;16(7):e0254610. doi: 10.1371/journal.pone.0254610 (PMC8279392; doi:10.1371/journal.pone.0254610)
Supplement: Attachment — Submitted filename: Response to reviewer.docx [file pone.0254610.s007.docx]

I Anna S Olsson, Ph.D.

Academic Editor

PLOS ONE

May 13, 2021

Dear Dr. I Anna S Olsson:

Thank you for inviting us to submit a revised draft of our manuscript entitled, “Prevalence and Risk Factors for Feather-damaging Behavior in Psittacine Birds: Analysis of a Japanese Nationwide Survey” to Journal of PLOS ONE. We also appreciate the time and effort you and each of the reviewers and editor have dedicated to providing insightful feedback on ways to improve our paper. Thus, it is with great pleasure that we resubmit our article for further consideration. We have incorporated changes that reflect the detailed suggestions that you graciously provided. We also hope that our changes and responses below satisfactorily address the issues and concerns that you raised.

To facilitate your review of our revisions, the following is a point-by-point response to the comments delivered in your letter dated May 9, 2021.

**Response to Additional Editor**

Thank you for your review of our paper. We have answered each of your points below.

1. Line 41 "higher risk factors for feather-damaging behavior than which other potential risk factors" is not grammatically correct - do you mean "higher risk factors for feather-damaging behavior than any other potential risk factors"?

**Response**: Thank you for correcting the grammar. We revised "higher risk factors for feather-damaging behavior than any other potential risk factors".

2. Line 54 This sentence begins with "Although the cause of FDB is believed to be psychological stress" and then you present a number of potential origins, of which many are indeed situations of psychological stress. "Although" is misleading in this context, as it suggests that the examples are of situations other than psychological stress. You can simply start the sentence with "The cause of FDB".

**Response**: Thank you for this suggestion. We revised to start the sentence with "The cause of FDB". We remove reference No.18. We renumbered the reference list.

3. Lines 73-75 This paragraph is overall about the aim of your study and what you collected data on. It is misleading to say that you focused on regional differences, as the comparison between countries is not a part of your study, but instead refers to a discussion of your results with those of other studies. Please remove this sentence.

**Response**: Thank you for this suggestion. We remove this sentence. Reference No.12–14 are cited elsewhere.

4. Lines 106-116 and 313-4: The way you refer to separation anxiety gives the impression that you asked the owner to observe the behaviour of the bird specifically in the situation of leaving home and returning home. Did you do this? Or did you simply ask the owner how the bird behaves when they leave home and when they return? If you did ask them specifically to observe behaviour to collect data, then your description is correct. If you asked them how the bird behaves but did not ask them to observe this in a specific way, you need to revise the wording.

**Response**: Thank you for this suggestion. We asked the owner how the bird behaves when they leave home and when they return. We revised this sentence to “The presence or absence of signs of separation anxiety was determined by the owner based on the behavior of the bird when the owner leaves home and the condition inside the cage when the owner returns home”.

**Section Limitations of the study**

5. Please reflect on the limitations of owner-reported data for behaviour. How reliably do you expect that owners can distinguish the different kinds of behaviour? I would argue that it is more difficult even for an experienced bird owner to recognise different kinds of behaviour and hence provide reliable data, than for them to provide reliable data for the other parameters you collected information on, so I would expect that there is more noise in the behaviour data.

**Response**: Thank you for this suggestion. We added the sentence “it may be more difficult even for an experienced bird owner to recognize different kinds of behavior such as stereotyped behavior, reproductive behavior, or signs of separation anxiety and provide reliable data, than for them to provide reliable data for the other parameters”.

**Response to reviewer #1**

Thank you for your review of our paper. We have answered each of your points below.

1. The paper is much improved. The one outstanding issue is that interactions are still handled incorrectly. When a model reveals a significant interaction, ADDITIONAL ANALYSES need to be run to investigate what drives it. An interaction means one of three things: an apparent effect is stronger in some sub-groups than others; an apparent effect is only present in some sub-groups; or an apparent effect flips direction in some sub-groups compared to others. The only way to find out what's happening is id to drill down into the data and run additional analyses using subsets (sorry).

**Response**: I apologize for using the inappropriate term "interaction". We used the phi coefficient or Cramer’s V to measure correlations between categorical variables where P < 0.05. Therefore, we revised the term "interaction" to "correlation". Since multivariate logistic regression was used to adjust the confounding of the variables, we think that sub-group analysis is not necessary. We apologize again for the confusion.

2. Also could I ask that when you supply the survey results as an SOM, you upload your values as they were BEFORE you pooled them into your (somewhat arbitrary) taxon groups? That way interested researchers could then see values broken up by true species (not genus or higher), which would be very useful for people interested in running meta-analyses.

**Response**: Thank you for this suggestion. We added a supplementary file with the true species and scientific names. We added the sentence “The true species before grouping is described in the S4 file” to Lines 127-128.

**Response to reviewer #2**

Thank you for your review of our paper.

**Reference list**

The reference number 18 has been retracted because line 54 has been deleted. Therefore, the reference list has changed. We reviewed reference list to ensure that it is complete and correct.

Again, thank you for giving us the opportunity to strengthen our manuscript based on your valuable comments and queries. We have worked hard to incorporate your feedback and hope that you find these revisions sufficient for accepting our submission.

Sincerely,

Kazumasa Ebisawa

Nihon University, College of Bioresource Sciences, Laboratory of Veterinary Physiology

1866 Kameino, Fujisawa, Kanagawa 252-0880, Japan

TEL: +81-466-84-3800

E-mail: yokohamabirdclinic1997@gmail.com
